# Myogenic program dysregulation is contributory to disease pathogenesis in spinal muscular atrophy

**DOI:** 10.1093/hmg/ddu142

**Published:** 2014-04-01

**Authors:** Justin G. Boyer, Marc-Olivier Deguise, Lyndsay M. Murray, Armin Yazdani, Yves De Repentigny, Céline Boudreau-Larivière, Rashmi Kothary

**Affiliations:** 1 Ottawa Hospital Research Institute, Regenerative Medicine Program, Ottawa, ON, CanadaK1H 8L6; 2 Department of Cellular and Molecular Medicine; 3 Department of Medicine, University of Ottawa, Ottawa, ON, CanadaK1H 8M5; 4 School of Human Kinetics, Laurentian University, Sudbury, ON, CanadaP3E 2C6

## Abstract

Mutations in the survival motor neuron (*SMN1*) gene lead to the neuromuscular disease spinal muscular atrophy (SMA). Although SMA is primarily considered as a motor neuron disease, the importance of muscle defects in its pathogenesis has not been fully examined. We use both primary cell culture and two different SMA model mice to demonstrate that reduced levels of Smn lead to a profound disruption in the expression of myogenic genes. This disruption was associated with a decrease in myofiber size and an increase in immature myofibers, suggesting that Smn is crucial for myogenic gene regulation and early muscle development. Histone deacetylase inhibitor trichostatin A treatment of SMA model mice increased myofiber size, myofiber maturity and attenuated the disruption of the myogenic program in these mice. Taken together, our work highlights the important contribution of myogenic program dysregulation to the muscle weakness observed in SMA.

## INTRODUCTION

Spinal muscular atrophy (SMA) is an autosomal recessive disorder characterized by the degeneration of α-motor neurons in the spinal cord and is a major leading genetic cause of infant deaths (1). The deletion, rearrangement or mutation of the disease causing gene, survival motor neuron 1 (*SMN1*), is the cause of SMA in over 95% of patients (2–4). There are two virtually identical copies of the *SMN* gene, a telomeric copy, *SMN1*, and a centromeric copy, *SMN2* (5). Compared with the *SMN1* gene, a single nucleotide substitution identified in *SMN2* has a profound functional impact as only 10% of *SMN2* translated products are full-length and stable (6). In contrast to humans, mice have a single *Smn* gene and the homozygous deletion of the gene leads to death early during preimplantation embryonic development (7). The addition of the human *SMN2* transgene onto the *Smn*-null background rescues the embryonic lethality but leads to a severe phenotype in mice (8). These mice, designated *Smn^−/−^*;*SMN2*, live up to 6 days before succumbing to the disease. We have previously generated an intermediate mouse model of SMA (*Smn^2B/−^*) that survives to 4 weeks of age due to slightly higher Smn protein levels than the severe model (9). The *2B* mutation consists of a three-nucleotide substitution in the exon splicing enhancer of exon 7 of the endogenous mouse *Smn* gene (10).

Although SMA is primarily considered as a motor neuron disease, the involvement of muscle in its pathogenesis has not been fully investigated. C2C12 myoblasts with reduced Smn protein display abnormal proliferation, aberrant myoblast fusion and malformed myotubes (11). The maturation of muscle is coordinated by the sequential expression of myogenic regulatory factors, namely myoblast determination 1 (MyoD), myogenin and the muscle-specific regulatory factor 4 (MRF4; also known as Myf6). MyoD is an early marker of myogenesis while myogenin and MRF4 are expressed later and mark muscle differentiation. During postnatal muscle development, a high proportion of paired box protein 7 (Pax7) positive satellite cells undergoes proliferation and differentiation, and contributes to muscle growth (12). The idea of delayed muscle development in mouse models of SMA has been suggested by several groups, however this has, for the most part, been proposed on the basis that myofibers do not appear to increase in size shortly after birth in severe mouse models of SMA (13,14). Whether skeletal muscle defects contribute to the SMA phenotype has only recently begun to be investigated. Much remains to be uncovered in diseased skeletal muscles including why they are smaller in SMA (15). Mechanisms underlying muscle weakness in SMA can be targeted therapeutically to increase muscle strength, regardless of whether they are attributed to intrinsic defects, or the consequence of motor neuron pathology.

Here, we show abnormal expression of the myogenic program in primary myoblasts from *Smn^2B/−^* mice and *in vivo*, where we demonstrate the presence of delayed skeletal muscle development independent of myofiber degeneration and muscle denervation in mouse models of SMA. The administration of the histone deacetylase (HDAC) inhibitor trichostatin A (TSA) ameliorated muscle maturity in SMA model mice. These data demonstrate that Smn is required for proper perinatal muscle development.

## RESULTS

### Decreased levels of myogenic regulatory factors and fusion defects in *Smn^2B/−^* primary myoblasts

**Figure 1.**
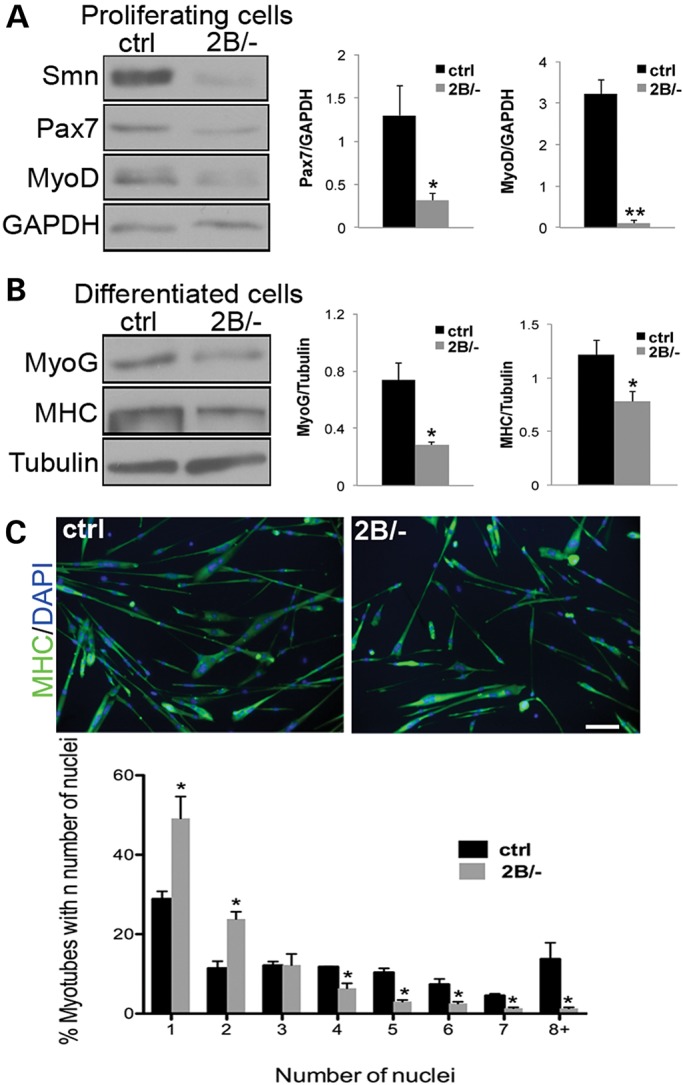
Altered myogenic program and aberrant myotube formation in primary cells from *Smn^2B/−^* mice. (**A**) Immunoblot analysis reveals decreased Smn, Pax7 and MyoD protein levels in proliferating primary myoblasts established from control and *Smn^2B/−^* mice. Bar graph showing a significant decrease in Pax7 and MyoD protein levels. (**B**) Immunoblot analysis and quantification reveals significantly decreased levels of myogenic differentiation markers myogenin (MyoG) and myosin heavy chain (MHC) in cultured primary cells differentiated for 5 days. (**C**) Representative images of control and *Smn^2B/−^* myoblasts differentiated into myotubes for 72 h and stained for MHC. Quantification of the number of nuclei per myotube revealed that there is a decrease in myoblast fusion in *Smn^2B/−^* cells compared with controls. Scale bar = 50 μm. *N* = 3 for all experiments, *, *P* < 0.05; **, *P* < 0.01.

To study myogenesis in a genetically relevant manner and in an intrinsic way by eliminating the negative contribution that motor neuron degeneration might have on muscle cells, we isolated primary myoblasts from *Smn^2B^*^/−^ mice. As expected, Smn protein levels were dramatically reduced in *Smn^2B/−^* myoblasts (Fig. 1A). Surprisingly, myoblasts under normal growth conditions displayed a 4-fold decrease in Pax7, and a 28-fold decrease in MyoD protein levels (Fig. 1A). To assess whether the mis-regulation of Pax7 and MyoD might have an impact on myoblast differentiation, cells were seeded at equal densities, grown to confluence and induced to differentiate by serum deprivation for 5 days. We observed a significant decrease (2.6-fold) in the level of myogenin, an early marker of myogenic differentiation, and a similar decrease (1.6-fold) in myosin heavy chain (MHC), a late marker of myogenic differentiation (Fig. 1B). To assess whether the aberrant expression of the myogenic program had any biological consequences, we examined the fusion potential of *Smn^2B^*^/−^ myoblasts. Upon differentiation, we observed a general decrease in fusion in *Smn^2B/−^* myotubes as evidenced by an increased number of mononucleated MHC positive cells and a corresponding decrease in myotubes with 8 or more nuclei in *Smn^2B/−^* myotubes compared with control myotubes (Fig. 1C). These results are reminiscent of those we have previously obtained using the hypomorphic Smn knockdown C2C12 cells (11). Together, our results suggest that in culture, myoblasts from *Smn^2B/−^* mice have differentiation and fusion defects.

### The myogenic program is delayed in skeletal muscle from mouse models of SMA

**Figure 2.**
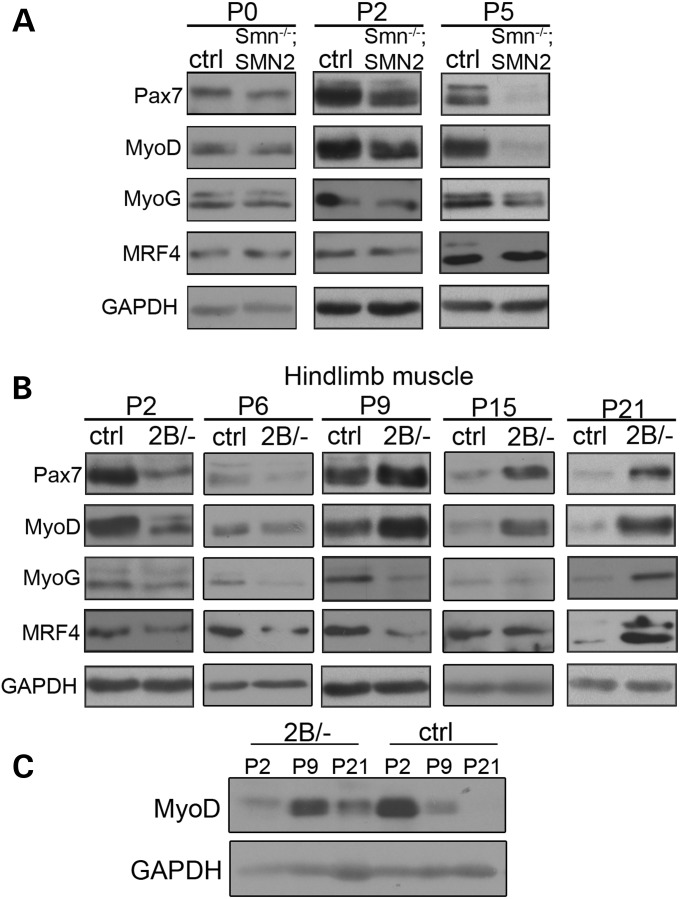
Delayed expression of myogenic proteins in mouse models of SMA. (**A**) Representative immunoblots in hindlimb muscle from P0, P2 and P5 *Smn^−/−^*;*SMN2* mice revealed a robust decrease over time in Pax7, MyoD and myogenin protein levels compared with controls. The expression of MRF4 was unchanged at all time points. (**B**) Representative immunoblots showing aberrant expression of myogenic proteins at different time points in *Smn^2B/−^* mice. The levels of myogenic factors are decreased in P2 muscles and increased at P21. At P6, MyoG protein levels were decreased. The levels of Pax7 and MyoD were high at P9 in *Smn^2B/−^* compared with controls, while those of MyoG were low. Only Pax7 expression was mis-regulated at P15 compared with controls. (**C**) Time-course analysis of MyoD protein levels reveals a delay in the peak expression from P2 in control samples to P9 in *Smn^2B/−^* muscle. *N* = 3 for all experiments.

Myogenic regulatory factors are normally highly expressed early during postnatal skeletal muscle development and their levels decrease significantly over time. By postnatal day (P) 21, these factors are difficult to detect relative to P2 (Supplementary Material, Fig. S1A). Interestingly, Smn protein expression from P2 to P21 follows a very similar trend suggesting a need for Smn early in muscle development (Supplementary Material, Fig. S1A). We assessed the expression of myogenic proteins at P0, P2 and P5 in hindlimb muscle from *Smn^−/−^*;*SMN2* and control mice (Fig. 2A and quantified in Supplementary Material, Fig. S2A). The protein levels of Pax7 in *Smn^−/−^*;*SMN2* mice were comparable to controls at P0 however, we observed a significant decrease at P2 and P5. Moreover, Pax7 transcripts were significantly decreased at P5 in *Smn^−/−^*;*SMN2* animals compared with control (Supplementary Material, Fig. S1B). The decreased levels of Pax7 at P2 and P5 translated to fewer Pax7 positive cells in skeletal muscles from *Smn^−/−^*;*SMN2* mice compared with controls (Supplementary Material, Fig. S3). At P0, no change in MyoD protein levels was detected and at P2 a trend towards decreased levels of MyoD was observed. Both protein and transcript levels of MyoD were significantly decreased at P5 in *Smn^−/−^*;*SMN2* mice compared with controls (Fig. 2A and Supplementary Material, Fig. S1C). Myogenin levels were unchanged at P0 and P2 in *Smn^−/−^*;*SMN2* mice. At P5 however, a significant decrease in myogenin mRNA and protein levels was observed compared with control counterparts (Fig. 2A and Supplementary Material, Fig. S1C). The level of MRF4 was unchanged at all time points assessed. Therefore, by P5, the levels of Pax7, MyoD and myogenin are lower in skeletal muscle from *Smn^−/−^*;*SMN2* mice compared with controls.

Immunoblot analysis was also performed on hindlimb skeletal muscle from *Smn^2B/−^* mice at early presymptomatic (P2 and P6), late presymptomatic (P9), and phenotype stages (P15 and P21) time points (Fig. 2B and quantified in Supplementary Material, Fig. S2). Similar to *Smn^−/−^*;*SMN2* mice, Pax7 levels were significantly decreased in *Smn^2B/−^* skeletal muscle at P2 compared with controls. No difference in Pax7 protein levels was detected at P6, however an increase in Pax7 levels was observed from P9 to P21 in *Smn^2B/−^* animals compared with controls (Fig. 2B). Furthermore, we observed increased Pax7 transcript levels and an increased proportion of Pax7 positive cells present in *Smn^2B/−^* skeletal muscle compared with controls at P21 (Supplementary Material, Figs S1C and S3D). Interestingly, we did not observe any difference in the proportion of M-cadherin positive cells in skeletal muscle from *Smn^2B/−^* compared with controls (Supplementary Material, Fig. S4). MyoD protein levels were significantly decreased at P2 and demonstrated a trend toward lower levels at P6 in *Smn^2B/−^* mice compared with controls. Similar to Pax7, MyoD levels were increased at P9 in skeletal muscle from *Smn^2B/−^* animals relative to controls (Fig. 2B). MyoD levels remained high up to P21 where significantly more MyoD mRNA and protein levels were observed in *Smn^2B/−^* mice compared with control mice (Fig. 2B and Supplementary Material, Fig. S1E). Myogenin protein levels decreased in *Smn^2B/−^* samples at P2 and remained low until P15 where levels were comparable to controls. By P21, myogenin protein levels were increased in *Smn^2B/−^* mice as were myogenin transcripts (Fig. 2B and Supplementary Material, Fig. S1E). The protein levels of MRF4 were significantly decreased at P2 and were not different than controls until P21 where the levels were increased in *Smn^2B/−^* mice (Fig. 2B). MRF4 transcripts were increased at P21 in *Smn^2B/−^* mice in agreement with the protein data (Supplementary Material, Fig. S1E). The delay in expression of the myogenic regulatory factors becomes even more apparent when control and *Smn^2B/−^* samples from P2, P9 and P21 are processed together on the same blot (Fig. 2C). Normally, the levels of MyoD decline dramatically from P2 onwards, however this phenomenon is delayed in the *Smn^2B/−^* muscle.

We have also extended our analysis to a third mouse model of SMA, the commonly used *Smn^−/−^*;*SMN2;Δ7* mouse. Using skeletal muscle lysate collected from phenotypic P13 *Smn^−/−^;SMN2;Δ7* and control mice, we show a decrease in the protein levels of Pax7, MyoD and myogenin, and to a lesser extent MRF4 (Supplementary Material, Fig. S5). Taken together, this data demonstrate that there is a significant disruption in the myogenic program an all three mouse models of SMA examined. In severe models (*Smn^−/−^*;*SMN2* and *Smn^−/−^*;*SMN2;Δ7*) this is evidenced by a dramatic decrease in the factors regulating myogenesis. In our milder model (*Smn^2B/−^*), this is evidenced by an early decrease in the levels of the factors followed by an increase at later stages of disease. We hypothesize that this difference is due to the more prolonged nature of the pathology in the milder models allowing myogenesis to ultimately proceed, albeit at a later time point.

The proper temporal expression of these myogenic transcription factors is essential for the subsequent expression of genes involved in muscle function. We investigated whether the aberrant expression of the myogenic program affected the expression of developmental MHC isoforms in muscles from SMA model mice. The expression of the embryonic and neonatal *MHC* transcripts was mis-regulated in a similar fashion as the myogenic regulatory factors in both mouse models of SMA (Supplementary Material, Fig. S6). That is, compared with control counterparts, a decrease in the mRNA expression of embryonic and neonatal *MHC* was detected in phenotype stage *Smn^−/−^*;* SMN2* mice, while these transcripts were increased in *Smn^2B/−^* samples at P21. These data suggest that the pathological reduction of Smn leads to muscles with altered levels of developmentally important myogenic genes.

### Increased immature myofibers in skeletal muscles of mouse models of SMA

**Figure 3.**
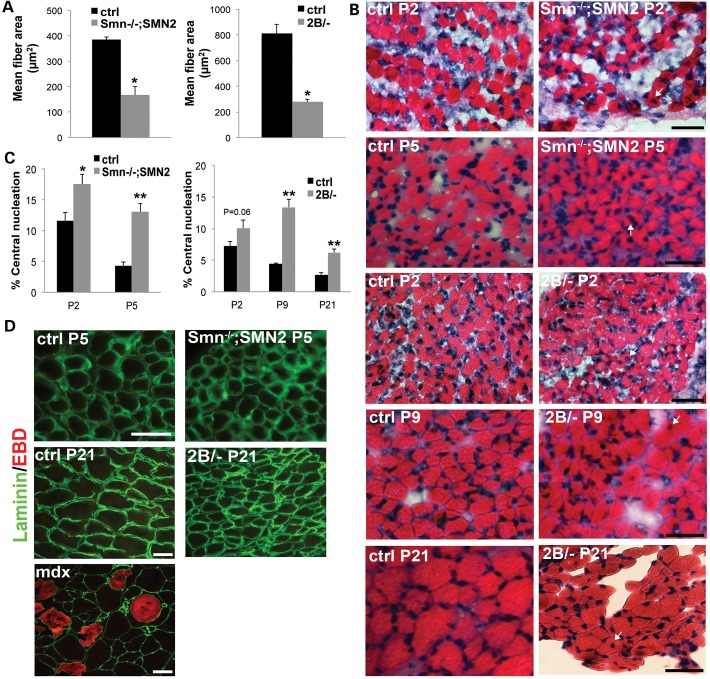
Decreased myofiber size and increased number of immature myofibers in muscle from mouse models of SMA. (**A**) Cross-sectional area measurements revealed smaller fibers in both mouse models at phenotype stage. Cross-sections of TA muscles from *Smn^−/−^*;*SMN2* and *Smn^2B/−^* mice were taken at P5 and P21 respectively. (**B**) Representative images of hematoxylin and eosin stained cross-sections from control, prephenotype P2 *Smn^−/−^*;*SMN2*, P2 and P9 *Smn^2B/−^* mice, and phenotype stage P5 *Smn^−/−^*;*SMN2* and P21 *Smn^2B/−^* TA muscles. Examples of myofibers with centrally located nuclei are depicted with arrows. Scale bars for P2 samples = 50 μm and scale bars for P5, P9 and P21 samples = 100 μm. (**C**) Quantification revealed an increased number of centrally located nuclei in both P2 and P5 *Smn^−/−^*;*SMN2* muscles. No change in the proportion of immature myofibers was observed at P2 in *Smn^2B/−^* mice however, an increase in myofibers with centrally localized nuclei was observed in P9 and P21 samples. (**D**) Evan's blue dye (EBD) is taken up by degenerating fibers and is detected as a red fluorescence signal. Representative images demonstrating the absence of EBD uptake in muscle of control and phenotypic *Smn^−/−^*;*SMN2* and *Smn^2B/−^* mice. TA muscle sections from the *mdx* muscular dystrophy mouse were used as a positive control for EBD uptake. Scale bar for P5 samples = 100 μm and the scale bar for P21 and *mdx* samples = 50 μm. *N* = 3 for all experiments. *, *P* < 0.05; **, *P* < 0.01.

Since our analysis of the myogenic program suggested a delay in skeletal muscle development in mouse models of SMA, we performed a histological analysis to assess the consequence of this molecular phenotype. The cross-sectional area of tibialis anterior (TA) myofibers from P5 *Smn^−/−^*;*SMN2* and P21 *Smn^2B^*^/−^ mice was significantly decreased compared with control (Fig. 3A). We also noted a higher proportion of myofibers with centrally located nuclei in both *Smn*^−/−^;*SMN2* and *Smn^2B^*^/−^ mice at phenotypic (P5 and P21 respectively) stages. Importantly, this was also seen at prephenotypic stages, demonstrating that centrally located nuclei are an early feature of disease pathogenesis (Fig. 3B and C).

Central nucleation is observed during muscle development and can also be a histological hallmark of muscle fiber regeneration following a bout of degeneration. To investigate whether this central nucleation was due to regeneration following degeneration, we performed the Evan's blue dye assay to assess whether skeletal muscles from mouse models of SMA have degenerating myofibers. We did not observe any degenerating fibers present in phenotype stage *Smn*^−/−^;*SMN2* and *Smn^2B^*^/−^ muscles (Fig. 3D). In contrast, muscle fibers from the *mdx* mouse model of muscular dystrophy display numerous examples of degeneration as evidenced by dye uptake (Fig. 3D). These data suggest that TA muscle fibers from mouse models of SMA are not undergoing degeneration, but rather that the increase in central nucleation observed is indicative of impairment in muscle development.

### The aberrant myogenic program correlates with muscle denervation but can occur independently of muscle denervation in mouse models of SMA

**Figure 4.**
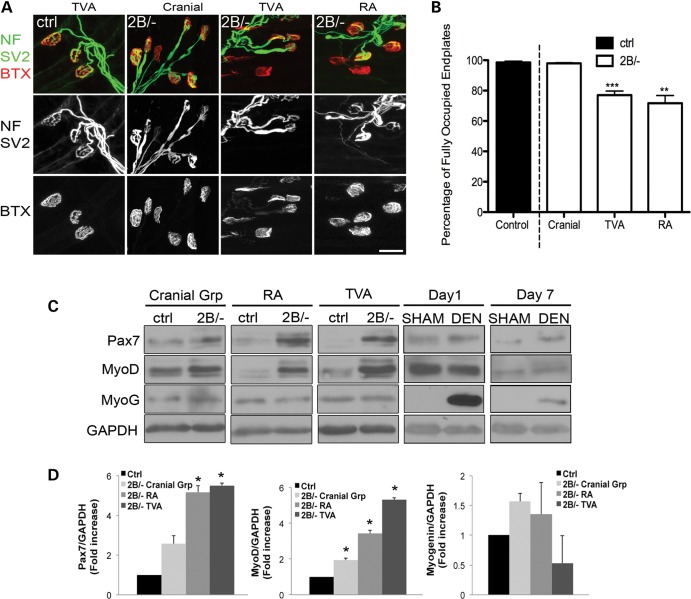
The myogenic program is altered even in muscles not subject to denervation in *Smn^2B/−^* mice. (**A**) Representative images showing fully intact NMJs from cranial, TVA and RA muscles of control and P21 *Smn^2B/−^* mice. Postsynaptic acetylcholine receptors were labeled with alpha-bungarotoxin (BTX, red) while the presynaptic terminal was labeled with antineurofilament (NF, green) and antisynaptic vesicle protein 2 (SV2, green). (**B**) Quantification of fully occupied endplates revealed differences between control and the TVA and RA muscles from *Smn^2B/−^* mice but not for cranial muscles, suggesting that the latter are not subject to denervation. (**C**) Representative immunoblots showing altered protein levels of Pax7 and MyoD in innervated (cranial) and denervated (RA and TVA) muscles of *Smn^2B/−^* mice. Interestingly, Pax7 and MyoD levels are unaffected in muscles following experimental denervation, at 1 or 7 days postsurgery. By comparison, a robust increase in myogenin levels can be detected as early as 1 day postdenervation and persists to 7 days following denervation. (**D**) Quantification analyses revealed differences in Pax7 and MyoD protein levels in all three muscle groups examined. The levels of myogenin however, were similar between control and *Smn^2B/−^* mice for innervated and denervated muscles. (MyoG = myogenin, DEN = denervated). Scale bar = 20 μm. *N* = 3 for all experiments. *, *P* < 0.05; **, *P* < 0.01, ***, *P* < 0.001.

The mis-regulation of the myogenic program in SMA model mice could be due in part to the innervation status of the muscle. To address this question, we have assessed the innervation status of various muscle groups namely, cranial muscles, the rectus abdominis (RA) muscle, and the transversus abdominis (TVA) muscle. Upon quantification of the percentage of fully occupied endplates, we observed that the cranial muscles appear fully innervated while the RA and the TVA are both muscles with pronounced denervation in P21 *Smn^2B/−^* mice (Fig. 4A and B). Therefore, we took advantage of our *Smn^2B/−^* mouse model in which there is differential vulnerability to denervation in various muscles, and assessed the levels of Pax7, MyoD and myogenin in these muscles at P21 phenotype stage. Pax7 and MyoD protein levels were significantly increased in all three *Smn^2B/−^* muscle types tested compared with controls (Fig. 4C and D). However, the degree to which Pax7 and MyoD levels were altered is much greater in the more denervated TVA and RA muscles (Fig. 4C and D). Interestingly, the level of myogenin was not statistically different between control and *Smn^2B/−^* muscles from the three groups despite muscle denervation being significantly present in the TVA and RA muscles (Fig. 4D).

To investigate whether Pax7 and MyoD levels could change following muscle denervation, we experimentally denervated wild-type mice. Interestingly, denervation did not impact on Pax7 and MyoD levels 1 and 7 days postdenervation but rather, caused a pronounced increase in myogenin levels as early as 1 day postdenervation (Fig. 4C). Thus, our results show that although the increase in Pax7 and MyoD correlates with the degree of denervation in *Smn^2B/−^* mice, severe denervation does not influence the levels of Pax7 and MyoD.

### Trichostatin A (TSA) rescues skeletal muscle defects in Smn^2B/−^ mice

**Figure 5.**
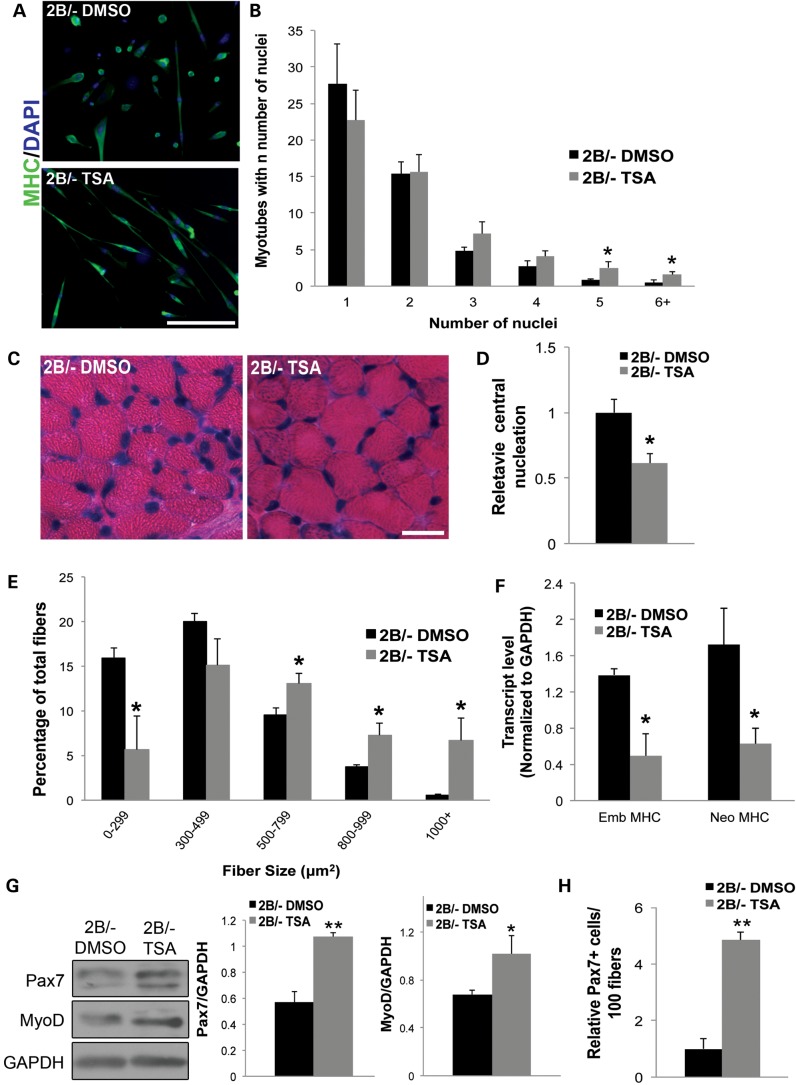
TSA treatment improves myoblast fusion and muscle maturity in *Smn^2B/−^* mice. (**A**) Representative images of *Smn^2B/−^* myotubes treated with either vehicle (DMSO) or TSA. Myoblasts were treated for 24 h and then differentiated for 72 h and stained for myosin heavy chain (green) and 4′,6-diamidino-2-phenylindole (blue). (**B**) Quantification revealed that TSA treated *Smn^2B/−^* cells had significantly more myotubes with five or more nuclei compared with *Smn^2B/−^* controls. (**C**) Representative images of hematoxylin and eosin stained cross-sections of TA muscle from P25 *Smn^2B/−^* mice treated with either DMSO or TSA. (**D**) Quantification revealed a significant decrease in the proportion of centrally located nuclei in *Smn^2B/−^* muscles treated with TSA relative to those treated with DMSO. (**E**) Histogram demonstrating significantly fewer small caliber fibers and an increased proportion of large caliber fibers in muscles of *Smn^2B/−^* animals following TSA treatment. (**F**) Quantification of RT-QPCR results demonstrating a significant decrease in transcript levels of embryonic (Emb) MHC and neonatal (Neo) MHC in P25 *Smn^2B/−^* mice treated with TSA compared with *Smn^2B/−^* mice treated with DMSO. (**G**) *Smn^2B/−^* mice were treated for 3 days with either DMSO or TSA starting at P3. Immunoblot analyses demonstrating an increase in the protein levels of Pax7 and MyoD in TSA treated P6 *Smn^2B/−^* compared with DMSO controls. (**H**) TSA treated *Smn^2B/−^* mice showed increase in Pax7 positive cell numbers compared with DMSO controls. *N* = 3 for all experiments. Scale bars = 100 μm. *, *P* < 0.05; **, *P* < 0.01.

Administration of the HDAC inhibitor TSA has previously been shown to extend survival in a severe mouse model of SMA. This improvement was associated with a modest increase in full-length SMN expression from the *SMN2* gene (16). Furthermore, TSA decreased the expression of atrogenes in skeletal muscles of SMA model mice, which likely contributed to the motor behavior improvements observed following treatment (16,17). Interestingly, TSA treatment is also beneficial in the *Smn^2B/−^* mouse model that does not harbor the *SMN2* transgene; however in this case there was no detectable increase in Smn. Furthermore, other studies have demonstrated that TSA treatment of C2C12 cells increased myoblast fusion, and that TSA can increase the expression of early myogenic regulatory factors (18,19). We therefore investigated whether TSA could increase myofiber size and ameliorate muscle maturity in *Smn^2B/−^* mice. We began by testing whether TSA could improve the fusion capacity of *Smn^2B/−^* primary myoblasts. To do so, we treated *Smn^2B/−^* myoblasts with TSA or vehicle [dimethyl sulfoxide (DMSO)] for 24 h and subsequently induced the cells to differentiate for 72 h. We observed a >3-fold increase in TSA treated cells with regards to the number of myotubes with 5 nuclei, and 6 nuclei or more (Fig. 5A and B). These results demonstrated that TSA can improve the fusion potential of *Smn^2B/−^* primary myotubes.

To test whether systemic administration of TSA could benefit the muscle defects in SMA model mice, we treated phenotypic *Smn^2B/−^* mice for 2 weeks with either vehicle or TSA via intraperitoneal injection from P12. Histological analyses of treated muscles revealed that TSA reduced the proportion of myofibers with centrally located nuclei (Fig. 5C and D). Moreover, *Smn^2B/−^* mice treated with TSA had significantly fewer small caliber myofibers and more large caliber myofibers compared with *Smn^2B/−^* mice given DMSO (Fig. 5C and E). TSA administration resulted in a significant decrease in the transcript levels of embryonic MHC and neonatal MHC isoforms suggesting that these muscles were further along the maturation process than those from *Smn^2B/−^* animals given DMSO (Fig. 5F). Finally, to assess whether TSA could increase early markers of myogenesis prior to any motor phenotype, we treated *Smn^2B/−^* animals with TSA from P3 to P6. Relative to DMSO controls, we observed a significant increase in Pax7 and MyoD protein levels following TSA administration (Fig. 5G). These results demonstrate that TSA can increase myogenic proteins early in SMA model mice prior to overt motor neuron pathology. Furthermore, the administration of TSA led to a further increase in the proportion of Pax7 positive cells in P21 *Smn^2B/−^* animals when compared with DMSO treated *Smn^2B/−^* mice (Fig. 5H).

## DISCUSSION

Previous studies have focused on uncovering the reasons for the motor neuron vulnerability in SMA and developing therapeutic approaches aimed at increasing SMN levels in motor neurons. In this study, we demonstrate that Smn deficiency leads to delayed postnatal skeletal muscle development in primary myoblasts from *Smn^2B/−^* mice and in multiple mouse models of SMA with varying phenotypes. Furthermore, we show that the administration of TSA on SMA model mice can ameliorate the defects in muscle development observed.

A delay in muscle development had previously been suggested based on morphological studies in mice (13,14). This led us to investigate whether the myogenic program was altered in muscles from mouse models of SMA. We show that the levels of Pax7, MyoD, myogenin and MRF4 were altered in both primary *Smn^2B/−^* myoblasts and in SMA model mice. The expression of these myogenic genes rapidly decreases over time during postnatal development as the myofibers mature. Interestingly, the expression of Smn in muscle follows a similar profile, as levels drop dramatically in P21 wild-type mice. This observation suggests that Smn is more likely to play an important role in muscle development rather than in muscle structure maintenance.

In *Smn^2B/−^* hindlimb muscles, levels of all myogenic proteins assessed were low at an early presymptomatic (P2) stage and increased at the phenotype stage (P21) when compared with controls. Immunoblot analyses performed on late presymptomatic muscle revealed a shift in levels of Pax7 and MyoD, as they were increased in P9 *Smn^2B/−^* mice while myogenin and MRF4, which are markers of differentiation, remained low. In our study, Pax7 and MyoD appear to be most affected and their mis-regulation is likely the reason for the aberrant expression of myogenin and MRF4. Our time-course analysis reveals that indeed the protein levels of MyoD peak at P9 and that the impaired muscle maturation is likely the result of this delay in expression. Decreased expression of Pax7 could be indicative of muscle differentiation initiation (20). However, our histological findings demonstrating the increased presence of immature myofibers in SMA model mice, and the results from our primary myoblast cultures also suggest otherwise.

We had hypothesized that muscle denervation was influencing the expression of the myogenic program, however given our results in which the myogenic program was mis-regulated in the cranial muscles, it is likely that this defect is an intrinsic phenomenon. Further supporting this notion is the fact that the myogenic defects can be observed well before motor neuron loss and denervation which occurs after P2 and P9 in *Smn^−/−^*;*SMN2* and *Smn^2B/−^* mice, respectively (21). However, it also remains equally possible that predegenerative changes in motor neurons or at the neuromuscular junction (NMJ) in SMA model mice could contribute to the changes observed in the muscle prior to those time points. We have also demonstrated that treatment with TSA can ameliorate muscle maturation in *Smn^2B/−^* mice. The effects of TSA on *Smn^2B/−^* primary myoblasts as well as on presymptomatic *Smn^2B/−^* animals demonstrate that this compound can have intrinsic benefits in skeletal muscle. Our data suggest that alterations of the myogenic program may be a reason for muscle vulnerability and that this defect can be targeted therapeutically. Regardless of whether the mis-regulation of the myogenic program is intrinsic or not, muscle defects are present in mouse models of SMA and can be targeted therapeutically to increase muscle function. Indeed, Martinez *et al*. (22) have demonstrated that using the Cre drivers *MyoD*^cre^ or *Myf5^cre^* to restore the expression of SMN in skeletal muscle or in motor neurons using the *ChAT^cre^* mouse led to comparable benefits in a mouse model of SMA. These results contrast previous findings in which the *HSA* promoter was used to drive expression of SMN in the skeletal muscle of SMA model mice (23). These latter mice did not show any significant benefit. However, it is likely that the HSA promoter is active only in myotubes and would not have imparted SMN expression in satellite cells or myoblasts. Thus, we believe that the skeletal muscle expression in the *HSA*-SMN mice would be too late during myogenesis to yield any significant phenotypic improvements in the SMA mice.

During the initial embryonic muscle formation, the first muscle fibers are generated and these serve as templates for a second wave of muscle formation in the perinatal phase (24). This second wave is characterized by the significant contributions of myogenic progenitors, which proliferate, differentiate and increase muscle size. In *Smn^−/−^*;*SMN2* skeletal muscle, it would appear that the level of Smn is too low at the required time point to fulfill its function(s) during the perinatal phase. In turn, this leads to a robust decrease in the myogenic program and to immature myofibers and muscle weakness. In *Smn^2B/−^* muscle however, our time-course analysis suggests that the myogenic program peaks at P9 instead of P0–P2 like in control mice. This delay ultimately has a profound impact on muscle morphology of *Smn^2B/−^* mice as we observed decreased cross-sectional myofiber area and increased immature myofibers. Therefore, in *Smn^2B/−^* mice the level of full-length Smn protein appears to be sufficient to allow the progression of skeletal muscle development albeit in a delayed manner.

In summary, the present study demonstrates early and persistent delayed muscle development in mouse models of SMA. The depletion of Smn hinders the normal maturation of skeletal muscle at the molecular and morphological levels, and likely contributes to the SMA phenotype. Future studies should focus on elucidating the functions of Smn during perinatal and early postnatal life, in particular addressing how Smn depletion leads to the mis-regulation of the myogenic program. The study of muscle in SMA research allows for a better understanding of the contributions of this tissue in the pathophysiology of the disease and highlights the importance of including muscle as a target tissue in therapeutic development.

## MATERIALS AND METHODS

### Mice and tissue dissections

In the present study, we took advantage of three different SMA mouse models, the *Smn^−/−^*;*SMN2* mice (Jackson Laboratories) that die around P6, the *Smn^−/−^*;*SMN2;Δ7* mice (Jackson Laboratories) that die at P13–P15, and the less severe *Smn^2B/−^* mice that die ∼4 weeks of age (8,9,25). The *Smn^2B/−^* mice were established in our laboratory and maintained in our animal facility on a C57BL/6 × CD-1 hybrid background. The *Smn^+/−^* mice (26) obtained from Jackson Laboratories were crossed with *Smn^2B/2B^* mice to produce *Smn^2B/−^* affected mice and *Smn^2B/+^* control littermates (9). The mice were housed and cared for according to the Canadian Council on Animal Care (CCAC) guidelines and the University of Ottawa Animal Care Committee protocols. Presymptomatic tissues were collected at P0 and P2 for severe *Smn^−/−^*;*SMN2* mice, and P2, P6 (early presymptomatic) and P9 (late presymptomatic) for the *Smn^2B/−^* mice. Tissues were collected from phenotypic stages at P5 in *Smn^−/−^*;*SMN2* mice, P13 in *Smn^−/−^*;*SMN2;Δ7* mice, and at P15 and P21 in *Smn^2B/−^* mice. Muscles used for RNA or protein extractions were flash frozen in liquid nitrogen, while muscles used for immunofluorescence and histology were embedded in optimum cutting temperature medium (Fisher) and frozen in cryomolds (Tissue-Tek). All tissues were stored at −80°C.

### TSA administration

*Smn^2B/−^*mice were treated with either TSA (10 mg/kg) or vehicle (DMSO) via intraperitoneal injections for 2 weeks starting at P12 following the appearance of the motor phenotype as previously described (16) for 2 weeks. To determine if TSA could increase myogenic protein expression in SMA model mice, *Smn^2B/−^* animals were treated from P3–P6 and skeletal muscles were collected and flash frozen for immunoblot analysis. In both experiments, samples were collected 2 h following the last injection.

### Hindlimb denervation

Denervation surgeries were performed in accordance with the guidelines set by the CCAC and the Laurentian University Animal Care Committee. Young adult mice were anesthetized by inhalation of isofluorane. The hindlimb muscles were bilaterally denervated by surgically exposing the sciatic nerve and removing a 2–3 mm segment of the nerve. This ensured that nerve transmission to hindlimb muscles was blocked and nerve regeneration was prevented. A sham procedure was performed as a control and consisted of exposing the mice to the same surgical procedures except for the sciatic nerve sectioning. Gastrocnemius muscles were collected and flash frozen from denervated and sham operated mice 1 and 7 days following surgery.

### Primary cell isolation and culture

*Smn^2B/−^* primary myoblasts were isolated from hindlimb muscles of 3-week-old mice as originally described (27). Cells were cultured on collagen-coated plates (Gibco) and maintained under growth conditions using Ham's F10 (Wisent) media supplemented with 20% fetal bovine serum, 2.5 ng/ml human recombinant basic fibroblast factor (Invitrogen), and 2% penicillin/streptomycin (Gibco). Primary myoblasts seeded at equal density were induced to differentiate with Dulbecco’'s Modified Eagle Medium (Wisent) supplemented with 5% horse serum (Gibco) and 1% penicillin/streptomycin. To assess the impact of TSA on fusion, *Smn^2B/−^* myoblasts were treated with 100 nm of TSA for 24 h and then induced to differentiate for 72 h as previously described (19). In all experiments, cells were maintained at 37°C with 5% CO_2_ and were provided fresh media every other day.

### Immunofluorescence

Cross-sections (10–12 μm) were collected using a cryostat (Leica) and fixed with 4% paraformaldehyde and then stained as previously described (28). A mouse-on-mouse kit (Vector Labs), and the primary antibodies targeting Pax7 (Developmental Studies Hybridoma Bank, DSHB) and M-cadherin (Santa Cruz) were used to highlight satellite cells while a laminin (Abcam) antibody was used to visualize the basal lamina. Antigen retrieval procedure consisting of an incubation of 30 min 95°C in sodium citrate buffer before blocking was used to enhance M-Cadherin labeling. Control and *Smn^2B/−^* primary myotubes were stained with myosin heavy chain (MHC) antibody (DSHB). All secondary antibodies were purchased from Molecular Probes and nuclei were visualized using 4′,6-diamidino-2-phenylindole (DAPI, Sigma). Immunofluorescence images were captured using a Zeiss Axioplan fluorescence microscope or a Zeiss Confocal microscope (LSM 510 Meta DuoScan). Pax7 positive cells were quantified from two different fields of view taken at 20× and expressed relative to the total number of myofibers present as assessed by laminin staining. The fusion capacity of primary myoblasts was determined by counting the number of nuclei per myotube using the ImageJ counting tool. For each sample, the data were averaged from five different fields of view taken at 10×. Immunomicrographs from *Smn^2B/−^* myotubes treated with DMSO or TSA were randomly scrambled and the results were blinded to the person counting.

### Evan's blue dye injection

Phenotype stage *Smn^−/−^*;*SMN2* and *Smn^2B/−^* mice were injected with Evan's blue dye (1%) diluted in phosphate buffered saline. The dye was administered by an intraperitoneal injection and at a concentration of 100 μl per 10 g of body weight for *Smn^2B/−^* mice, and 50 μl per 10 g of body weight for *Smn^−/−^*;*SMN2* mice. Tissues from *Smn^2B/−^* mice were harvested 24 h postinjection and 6 h postinjection for *Smn^−/−^*;*SMN2* mice. Skeletal muscles were sectioned using a cryostat, fixed with ice-cold acetone at −20°C for 10 min, and stained with laminin to visualize the myofibers. Degenerating fibers could be detected by the presence of a red signal using a fluorescence microscope. *mdx* mice, a model of Duchenne Muscular Dystrophy, were injected at phenotype stage with Evan's blue dye in parallel and served as a positive control for the presence of degenerating myofibers.

### Reverse-transcription

Flash frozen hindlimb muscle samples were homogenized and RNA was isolated using a column-based technique with the RNeasy kit (Qiagen) as previously described (28). RNA concentrations were obtained for each sample using a Nanophotometer spectrophotometer (MBI Lab Equipment). Samples were treated with DNase (gDNA wipeout buffer, Qiagen) to eliminate any potential DNA contamination. RNA was reverse transcribed using the Quantitect reverse transcription kit (Qiagen) as per the manufacturer's protocol.

### Histological analyses

Histological analyses were performed on hematoxylin and eosin stained cross-sections (10 μm) from frozen TA muscles of P2 and P5 *Smn^−/−^*;*SMN2*, and P2, P9 and P21 *Smn^2B/−^* mice. The TA muscle was selected as it is spared from histological denervation in mouse models of SMA and therefore it would likely not be influenced by muscle denervation induced atrophy (29,30). Sections were stained with hematoxylin and eosin using a standard protocol, images taken with a Zeiss Axioplan2 microscope, and the centrally located nuclei were counted using ImageJ software (NIH). The centrally located nuclei data are presented relative to the total number of myofibers present in a given field of view. Images to assess central nucleation were taken at 20× for P5, P9, P21 and the TSA data, and at 40× for P2 data. The cross-sectional area of the TA muscle from *Smn^2B/−^* mice treated with either DMSO or TSA was calculated by tracing the contour of myofibers using the tracing tool in ImageJ. A minimum of 200 myofibers were traced. Cross-sections from control and SMA model mice were collected from the same area of the TA muscle.

### Quantitative polymerase chain reaction (QPCR)

QPCR was performed to assess the mRNA levels of myogenic transcription factors such as paired box protein 7 (Pax7), myoblast determination 1 (MyoD), myogenin and the muscle-specific regulatory factor 4 (MRF4; also known as Myf6) in skeletal muscles from control and mutant mice. Each QPCR was performed in technical triplicate using primers described in Supplementary Material, Table S1. Each QPCR reaction contained 50 ng of cDNA, 2× SyBR Green JumpStart Taq ReadyMix for QPCR (Sigma-Aldrich), RNase/DNase-free water and appropriate primers (200 nm) in a final volume of 25 μl. Each reaction was heated at 94°C for 3 min followed by 40 cycles of denaturing at 94°C for 30 s, annealing (Supplementary Material, Table S1) for 30 s and elongation at 72°C for 30 s. To confirm amplicon specificity and size, a melting curve analysis was performed and the final QPCR products were migrated on a 2% agarose gel for each primer set. Two negative controls were included in every QPCR plate and consisted of water in lieu of cDNA and RNA free cDNA. QPCR results from each plate were generated from a standard curve with known amounts of cDNA. Quantified results were normalized to glyceraldehyde 3-phosphate dehydrogenase (GAPDH) transcript levels to control for loading.

### Protein analysis

Protein concentrations were determined using the Bradford method. Immunoblot analysis was performed as previously described (31). Primary antibodies used were: GAPDH (Abcam), MHC (MF20), MRF4 (Santa Cruz), MyoD (BD Transduction Laboratories), myogenin (BD Transduction Laboratories), Pax7, Smn (BD Transduction Laboratories) and tubulin (DSHB). Densitometric analyses were performed using ImageJ software. Immunoblot data are expressed as ratios using GAPDH or tubulin (for the differentiated myotube data) as a loading control for normalization.

### Statistical analyses

Data are presented as the mean ± standard error of the mean. A Student's *t* test was performed using Excel to compare the means. Significance was set at *P* < 0.05.

## SUPPLEMENTARY MATERIAL

Supplementary Material is available at *HMG* online.

## FUNDING

This project was funded by grants from the Canadian Institutes of Health Research (CIHR) and the Muscular Dystrophy Association (USA) to R.K. J.G.B. was supported by a Frederick Banting and Charles Best CIHR Doctoral Research Award, and L.M.M. by a Multiple Sclerosis Society of Canada Postdoctoral Fellowship. R.K. is a recipient of a University Health Research Chair from the University of Ottawa. Funding to pay the Open Access publication charges for this article was provided by the University of Ottawa.

## Supplementary Material

Supplementary Data
