# Supplementary material for: Myogenic program dysregulation is contributory to disease pathogenesis in spinal muscular atrophy
Source: Hum Mol Genet. 2014 Apr 1;23(16):4249–59. doi: 10.1093/hmg/ddu142 (PMC4103674; doi:10.1093/hmg/ddu142)
Supplement: Supplementary Data [file supp_23_16_4249__index.html]

Myogenic program dysregulation is contributory to disease pathogenesis in spinal muscular atrophy — Myogenic program dysregulation is contributory to disease pathogenesis in spinal muscular atrophy — Supplementary Data 

# Myogenic program dysregulation is contributory to disease pathogenesis in spinal muscular atrophy

## Supplementary Data

Supplementary Data

**Files in this Data Supplement:**

- Supplementary Data - Pdf file
