# Supplementary material for: Myogenic program dysregulation is contributory to disease pathogenesis in spinal muscular atrophy
Source: Hum Mol Genet. 2014 Apr 1;23(16):4249–59. doi: 10.1093/hmg/ddu142 (PMC4103674; doi:10.1093/hmg/ddu142)
Supplement: Supplementary Data [file supp_ddu142_ddu142supp.pdf]

Myogenic program dysregulation is contributory to disease pathogenesis in spinal muscular atrophy

Justin G. Boyer[1,2], Marc-Olivier Deguise[1,2], Lyndsay M. Murray[1], Armin Yazdani[1,2], Yves De Repentigny[1], Céline Boudreau-Larivière[3], and Rashmi Kothary[1,2,4#]

Supporting Information

SI Results

Figure S1. Mis-regulated myogenic program expression in mouse models of SMA.

(A) Time-course analysis of myogenic program expression in wild type hindlimb protein samples. The protein levels of Pax7, MyoD, myogenin, MRF4 and Smn were assessed at P2, P5, P9 and P21 by immunoblot and show a robust decrease during postnatal muscle development. (B) RT-QPCR was performed with RNA extracted from hindlimb muscle from phenotypic SMA model mice. A decrease in the level of *Pax7* transcripts was observed in severe P5 phenotype stage *Smn*$^{-/-}$*;SMN2* mice. (C) Analysis of expression revealed a significant decrease in *MyoD* and *myogenin* transcript levels in P5 hindlimb samples from *Smn*$^{-/-}$*;SMN2* mice compared to controls. (D) Levels of *Pax7* transcripts were increased in P21 phenotype stage *Smn*$^{2B/-}$ mice compared to controls. (E) *MyoD, myogenin, MRF4* transcript levels were increased in P21 *Smn*$^{2B/-}$ muscle samples compared to controls. N = 5 for all experiments. *, $p < 0.05$; **, $p < 0.01$. (Myogenin, MyoG).

Figure S2. Altered expression of the myogenic program in mouse models of SMA.

(A) Quantification of immunoblot analyses for the *Smn*$^{-/-}$*;SMN2* mouse model. We observed a 19% decrease in Pax7 levels at P2 and a 72% decrease in Pax7 levels at P5 in *Smn*$^{-/-}$*;SMN2* model mice. In P5 *Smn*$^{-/-}$*;SMN2* skeletal muscle, MyoD protein levels were decreased by 64% and those of myogenin were down by 69%. (B) Graphs showing the quantification results of immunoblots assessing the expression of the myogenic program in *Smn*$^{2B/-}$ mice. In *Smn*$^{2B/-}$ mice, Pax7 protein levels were down 47% at P2, however we observed a 60% increase in Pax7 levels at P9, a 178% increase at P15 and a 509% increase at P21. We demonstrate a 49% decrease in MyoD expression in P2 *Smn*$^{2B/-}$ mice. We observed a 47% and a 350% increase in MyoD protein levels in P9 and P21 *Smn*$^{2B/-}$ mice respectively. In *Smn*$^{2B/-}$ mice, myogenin protein levels were decrease by 30% at P2, 67% at P6 and 82% at P9. At P21 in *Smn*$^{2B/-}$ mice, myogenin protein levels were 526% higher than controls. MRF4 protein expression was 25% lower at P2 and 574% higher at P21 in *Smn*$^{2B/-}$ mice compared to controls. *, $p < 0.05$; **, $p < 0.01$. (Myogenin, MyoG).

Figure S3. Altered number of Pax7 positive cells in TA muscles from SMA model mice.

(A) Representative immunofluorescence images of pre-phenotype P2 *Smn*$^{-/-}$*;SMN2*, P2 *Smn*$^{2B/-}$, phenotypic P5 *Smn*$^{-/-}$*;SMN2*, P21 *Smn*$^{2B/-}$ and control TA muscles showing laminin (red), nuclei (DAPI, blue), and Pax7 (green) staining. (B) Bar graph demonstrating a 47% decrease in the number of Pax7 positive cells in severe P2 *Smn*$^{-/-}$*;SMN2* muscle compared to control samples. No statistically significant change was observed between P2 *Smn*$^{2B/-}$ and control samples, although a trend towards a decrease was noted. (C) Bar graph showing 57% fewer Pax7 positive cells in severe P5 *Smn*$^{-/-}$

;SMN2 muscle compared to controls. As well, quantification revealed a 47% increase in Pax7 positive cells in P21 *Smn*$^{2B/-}$ TA muscles compared to control. N = 3 for all experiments and scale bar = 10 µm. *, $p < 0.05$; **, $p < 0.01$.

Figure S4. The proportion of M-cadherin positive cells is unchanged in *Smn*$^{2B/-}$ mice compared to wild type.

(A) Representative immunofluorescence images from P21 *Smn*$^{2B/-}$ and control TA muscle samples. (B) Quantification of M-cadherin positive cells revealed comparable numbers of these cells in *Smn*$^{2B/-}$ mice and controls. N = 4 and scale bar = 50 µm.

Figure S5. Altered expression of the myogenic program in *Smn*$^{-/-}$*;SMN2;Δ7* mice.

Immunoblot analysis revealed a decrease in the protein levels of Pax7, MyoD, myogenin and to a lesser extent MRF4. Analysis was performed using hindlimb skeletal muscle from three independent phenotype stage P13 *Smn*$^{-/-}$*;SMN2;Δ7* mice. (Myogenin, MyoG).

Figure S6. Altered mRNA expression of MHC isoforms in SMA model mice.

RT-QPCR analysis was performed on RNA extracted from phenotype stage *Smn*$^{-/-}$*;SMN2* and *Smn*$^{2B/-}$ skeletal muscle. Changes in the levels of transcripts of the embryonic (Emb MHC) and neonatal (Neo MHC) myosin heavy chain isoforms in *Smn*$^{-/-}$*;SMN2* (A) and *Smn*$^{2B/-}$ (B) mice compared to controls. N = 5 for all experiments. *, $p < 0.05$; **, $p < 0.01$. (Myogenin, MyoG).

Table S1. Primers used for Quantitative Real-Time Polymerase Chain Reaction

**Targets**	**Sense (Forward) primer 5’-3’**	**Antisense (Reverse) primer 5’-3’**	**T_a**
Gapdh	TGAAGGGGTCGTTGATGG	AAAATGGTGAAGGTCGGTGT	62
Pax7	GACGACGAGGAAGGAGACAA	CGGGTTCTGATTCCACATCT	62
MyoD	TGGCATGATGGATTACAGCG	CCACTATGCTGGACAGGCAGT	62
MyoG	CATCCAGTACATTGAGCGCCTACA	AGCAAATGATCTCCTGGGTTGGGA	59
MRF4	CAAGCAAGAAATTCTTGAGGG	CGTTCCTCTGAAGAAATACTGTC	63
Neonatal MHC	TCGCTGGCTTTGAGATCTTT	ACGAACATGTGGTGGTTGAA	62
Embryonic MHC	GCAGATTCAGAAACTGGAGAC	CCTTAACACGCCGCTCATAC	62

T_a: annealing temperature

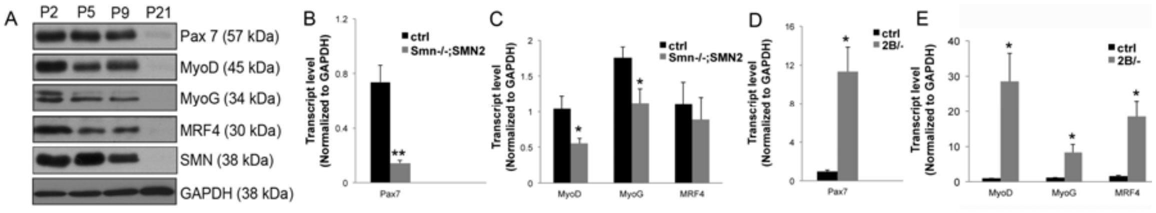

A
P2 P5 P9 P21
Pax 7 (57 kDa)
MyoD (45 kDa)
MyoG (34 kDa)
MRF4 (30 kDa)
SMN (38 kDa)
GAPDH (38 kDa)
B
Transcript level (Normalized to GAPDH)
ctrl
Smn-/-;SMN2
Pax7
C
Transcript level (Normalized to GAPDH)
ctrl
Smn-/-;SMN2
MyoD
MyoG
MRF4
D
Transcript level (Normalized to GAPDH)
ctrl
2B/-
Pax7
E
Transcript level (Normalized to GAPDH)
ctrl
2B/-
MyoD
MyoG
MRF4

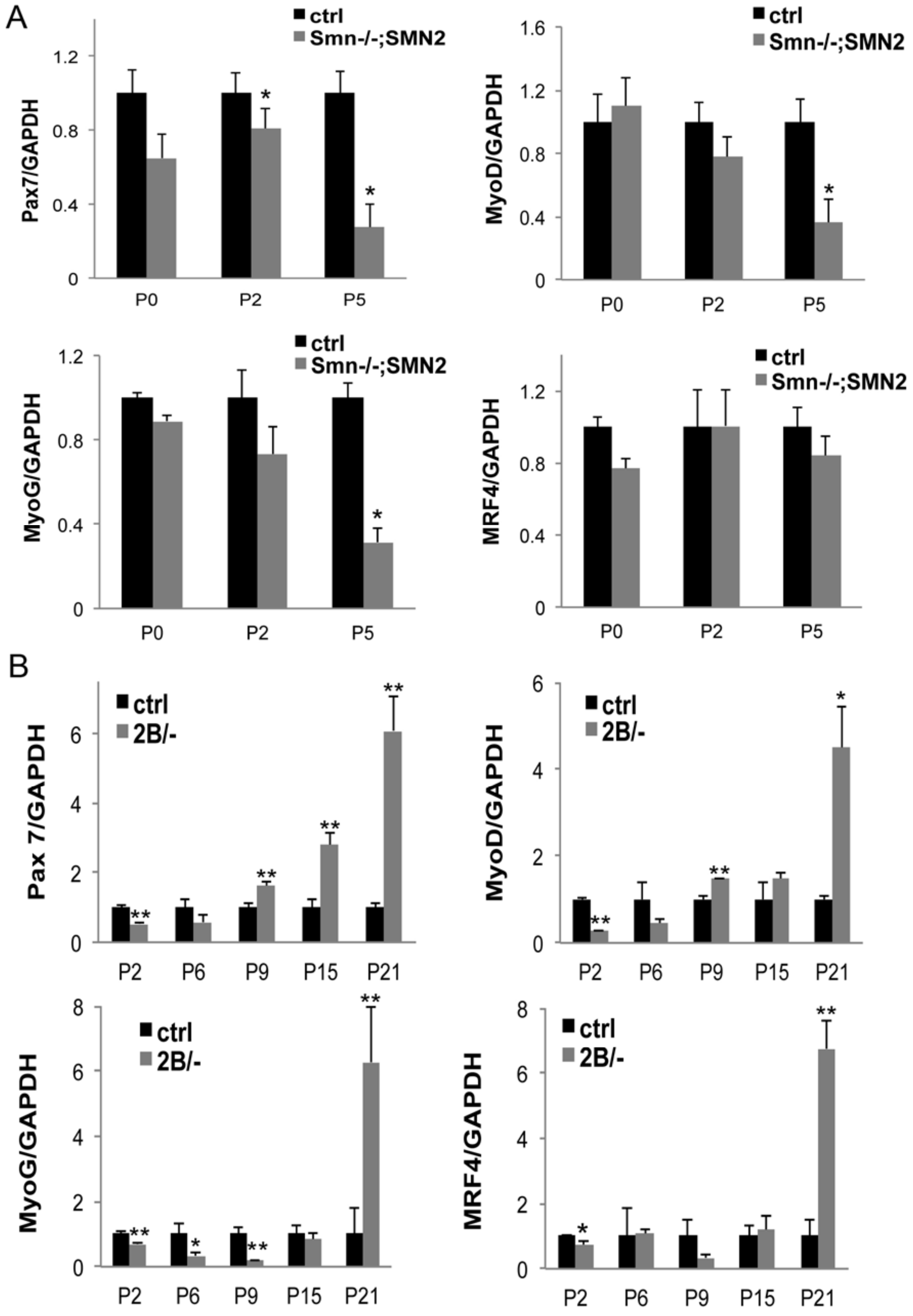

A
ctrl
Smn-/-;SMN2
Pax7/GAPDH
MyoD/GAPDH
MyoG/GAPDH
MRF4/GAPDH
P0
P2
P5
B
ctrl
2B/-
Pax 7/GAPDH
MyoD/GAPDH
MyoG/GAPDH
MRF4/GAPDH
P2
P6
P9
P15
P21

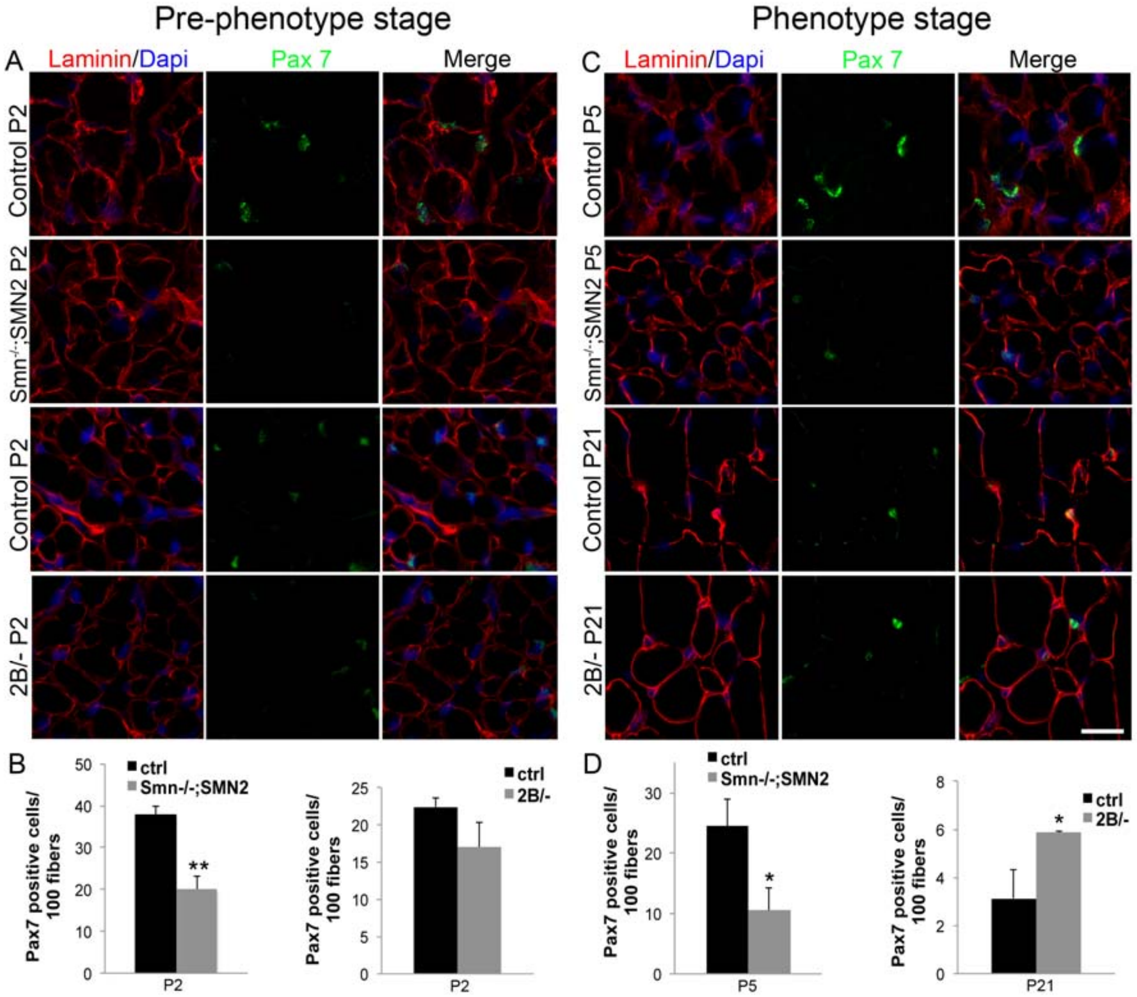

Pre-phenotype stage
Phenotype stage
A
Laminin/Dapi
Pax 7
Merge
Control P2
Smn-/-;SMN2 P2
Control P2
2B/- P2
C
Laminin/Dapi
Pax 7
Merge
Control P5
Smn-/-;SMN2 P5
Control P21
2B/- P21
B
Pax7 positive cells/ 100 fibers
ctrl
Smn-/-;SMN2
**
P2
ctrl
2B/-
P2
D
ctrl
Smn-/-;SMN2
*
P5
ctrl
2B/-
*
P21

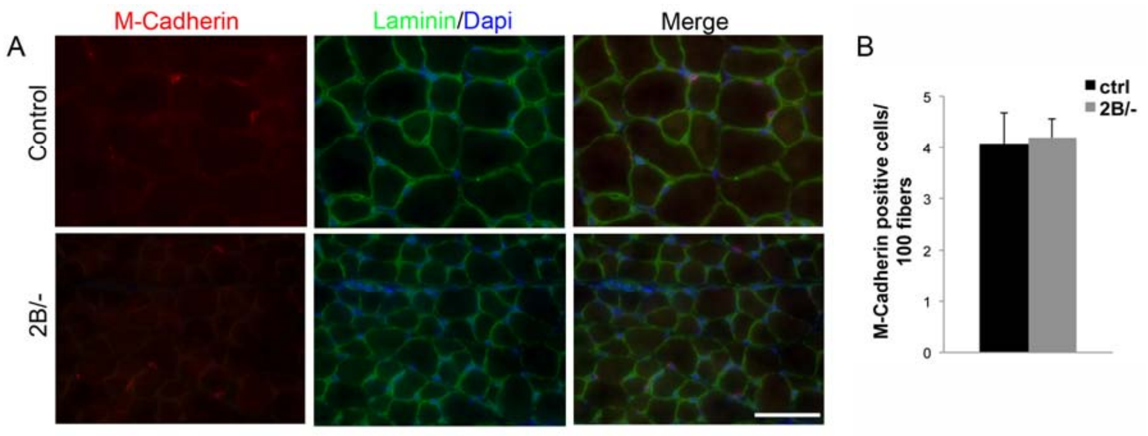
A
M-Cadherin
Laminin/Dapi
Merge
Control
2B/-
B
M-Cadherin positive cells/
100 fibers
0
1
2
3
4
5
ctrl
2B/-

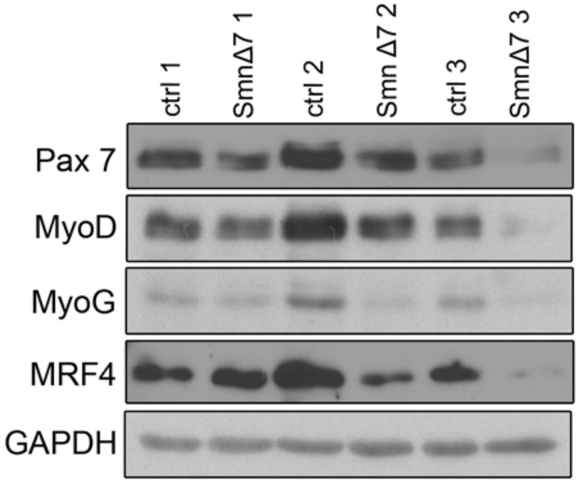
ctrl 1
SmnΔ7 1
ctrl 2
Smn Δ7 2
ctrl 3
SmnΔ7 3
Pax 7
MyoD
MyoG
MRF4
GAPDH

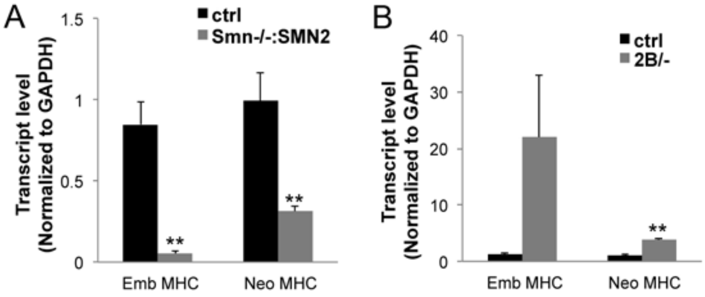
A
B
ctrl
Smn-/-:SMN2
2B/-
Transcript level (Normalized to GAPDH)
1.5
1
0.5
0
40
30
20
10
**
Emb MHC
Neo MHC
